# Sirolimus-eluting cobalt–chrome alloy stent suppresses stent-induced tissue hyperplasia in a porcine Eustachian tube model

**DOI:** 10.1038/s41598-022-07471-2

**Published:** 2022-03-02

**Authors:** Jeon Min Kang, Song Hee Kim, Yeon Joo Choi, Yubeen Park, Dae Sung Ryu, Woo Seok Kang, Jung-Hoon Park, Hong Ju Park

**Affiliations:** 1 Biomedical Engineering Research Center, Asan Institute for Life Sciences, Asan Medical Center, 88 Olympic-ro 43-gil, Songpa-gu, Seoul, 05505 Republic of Korea; 2 Department of Otorhinolaryngology-Head and Neck Surgery, Asan Medical Center, University of Ulsan College of Medicine, 88 Olympic-ro 43-gil, Songpa-gu, Seoul, 05505 Republic of Korea

**Keywords:** Biomedical engineering, Drug delivery, Preclinical research, Translational research

## Abstract

Various preclinical studies with developed Eustachian tube (ET) stents are in progress but have not yet been clinically applied. ET stent is limited by stent-induced tissue hyperplasia in preclinical studies. The effectiveness of sirolimus-eluting cobalt–chrome alloy stent (SES) in suppressing stent-induced tissue hyperplasia after stent placement in the porcine ET model was investigated. Six pigs were divided into two groups (i.e., the control and the SES groups) with three pigs for each group. The control group received an uncoated cobalt–chrome alloy stent (*n* = 6), and the SES group received a sirolimus-eluting cobalt–chrome alloy stent (*n* = 6). All groups were sacrificed 4 weeks after stent placement. Stent placement was successful in all ETs without procedure-related complications. None of the stents was able to keep its round shape as original, and mucus accumulation was observed inside and around the stent in both groups. On histologic analysis, the tissue hyperplasia area and the thickness of submucosal fibrosis were significantly lower in the SES group than in the control group. SES seems to be effective in suppressing stent-induced tissue hyperplasia in porcine ET. However, further investigation was required to verify the optimal stent materials and antiproliferative drugs.

## Introduction

Eustachian tube (ET) has essential functions in the middle ear (e.g., ventilation, protection against pathogenic microorganisms, and secretion transport into the nasopharynx)^1^. Protection from nasopharyngeal sound and reflux is also included^2^. ET is usually closed but it opens when swallowing, yawning, or chewing. However, ET dysfunction may occur when the tube does not open or close appropriately^3,4^. Dilatory (obstructive) ET dysfunction inhibits ET functions, and acute or chronic otitis media may develop if these functions cannot be preserved, which is one of the most common disorders in otolaryngology practice^5^. Current ET dysfunction treatments (e.g., nasal surgery, ventilation tube insertion, and pharmacologic agents) have been applied in patients. However, these treatments were limited in their effectiveness and may result in ET obstruction, infection, and permanent perforation of the tympanic membrane^3,6,7^. Balloon Eustachian tuboplasty has been introduced as an alternative dilatory ET dysfunction treatment^8^. Some patients fail to respond to dilation although several studies since 2010 have reported that balloon Eustachian tuboplasty is superior to conventional ET dysfunction treatment^8–11^. Thus, stent placement can serve as an effective therapeutic option^12,13^. Despite many ongoing preclinical studies that evaluate the technical feasibility and tissue response after stent placement in ET, stent-induced tissue hyperplasia caused by mechanical injury still represents a significant postprocedural complication^14–19^. Drug-eluting stents loaded with antiproliferative agents are believed to improve this situation.

**Figure 1:**
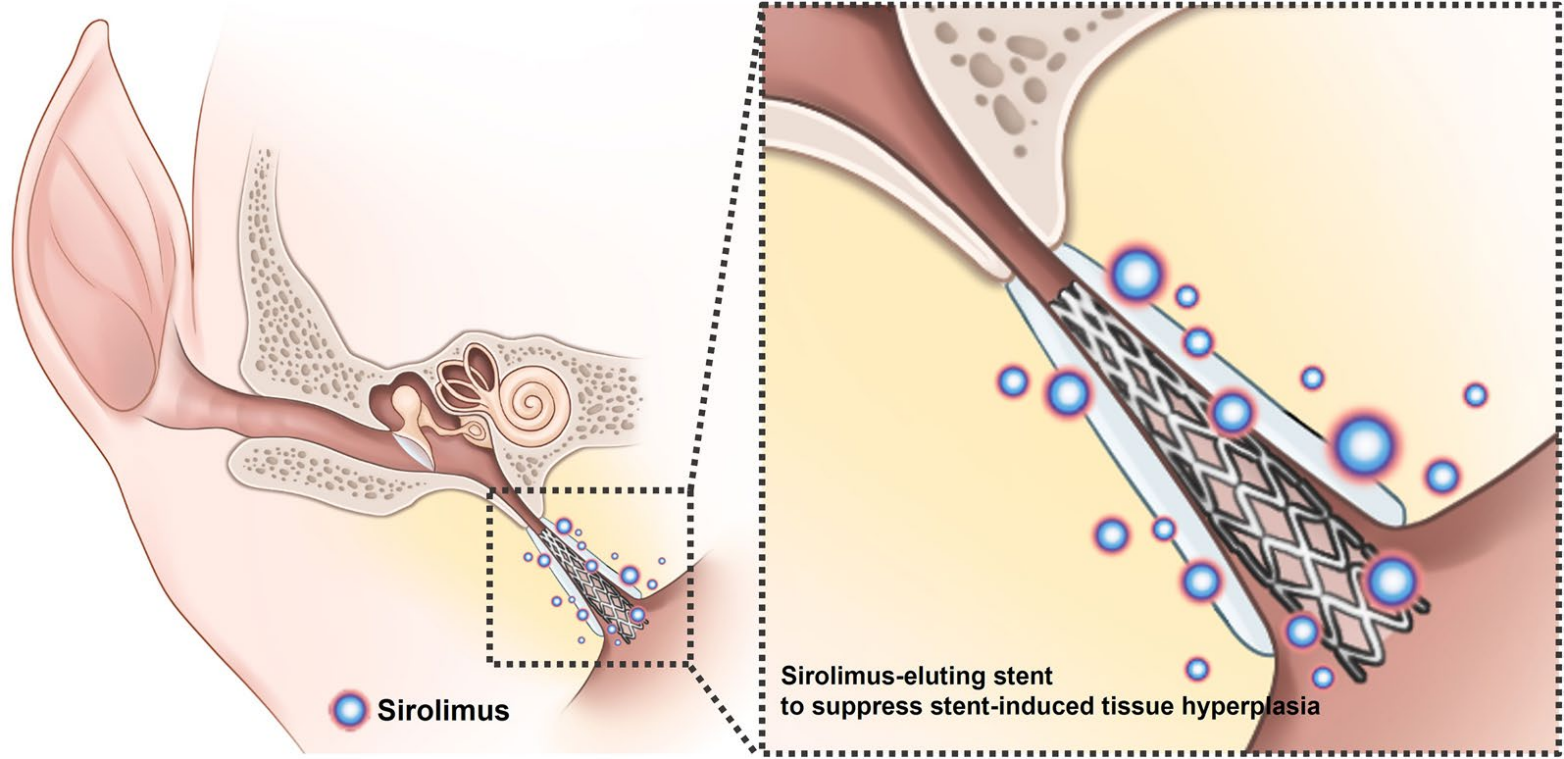
Schematic illustration of a sirolimus-eluting cobalt–chrome alloy stent (SES) for Eustachian tube dysfunction image showing sirolimus-eluting stent suppressed stent-induced tissue hyperplasia.

Drug-eluting stents have been used to suppress in-stent restenosis caused by tissue and neointimal hyperplasia after stent placement^20–23^. In general, stent struts or stent-covering membranes are coated with pharmacologic agents (e.g., everolimus, paclitaxel, and sirolimus)^20,23,24^. Sirolimus, which is a representative drug for antiproliferation, can inhibit several phases of the restenosis cascade (e.g., inflammation, neointimal hyperplasia formation, and collagen synthesis)^25^. Therefore, this study hypothesized that sirolimus-coated stent may prevent stent-induced tissue hyperplasia in the porcine ET (Fig. 1). This study aims to investigate the effectiveness of sirolimus-eluting cobalt–chrome alloy stent (SES) in suppressing stent-induced tissue hyperplasia after stent placement in the porcine ET model.

## Methods

### Preparation of sirolimus-eluting stent

**Figure 2:**
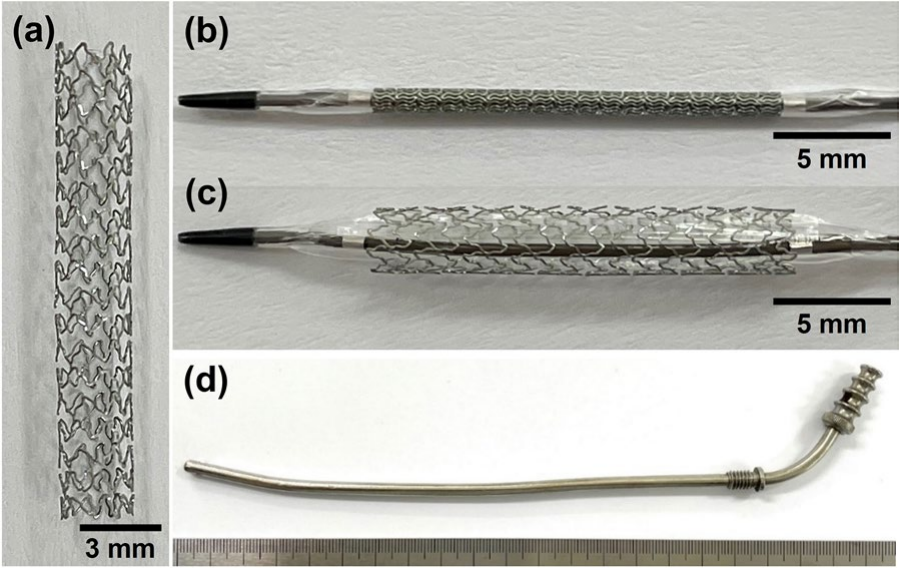
Cobalt–chrome (Co–Cr) alloy stent and metallic guiding sheath for Eustachian tube stent placement. Photograph showing (**a**) Co–Cr alloy stent and (**b**) balloon catheter crimped with a stent. (**c**) The balloon catheter is fully expanded with the stent. (**d**) The metallic guiding sheath was developed for the porcine Eustachian tube model.

The cobalt–chrome (Co–Cr) alloy stent was manufactured by laser cutting a Co–Cr alloy tube (Genoss Co., Ltd., Suwon, Korea). The stent platform featured open-cell two links with a uniform architecture that permits a high level of flexibility with optimal radial force, shortening, and conformability. The stent was 3 mm in diameter and 18 mm in length with a strut thickness of 78 µm (Fig. 2a). The Co–Cr alloy stent size was determined based on our previous study^19^.

Sirolimus was coated on the stent surface using the ultrasonic spray technique. SES is designed to release nearly 70% of its initial drug payload (1.15 µg/mm^2^) within the first 30 days following placement. A 3-µm ultrathin coating is applied only to the abluminal side of the stent to attain the desired drug release profile and minimize the polymer amount; this biodegradable coating comprises a proprietary blend of poly (lactic-co-glycolic acid) and poly (l-lactic acid)^26,27^. The Co–Cr alloy stent was crimped onto a 3-mm (diameter) and 28-mm (length) balloon catheter (Genoss Co., Ltd.; Fig. 2b). The stents are commercially available for coronary artery diseases in Korea.

### Metallic guiding sheath

A newly developed metallic guiding sheath for use in the porcine ET model was made of stainless steel (Fig. 2c). This sheath had an inner and outer diameter of 2 and 2.5 mm, respectively, and a total length of 250 mm. The distal 30 mm of the sheath was curved into a J shape at a 15° angle to the axis to enable easy access from the nose to the nasopharyngeal orifice of the ET in the porcine model.

### Animal study design

This study was approved by the Institutional Animal Care and Use Committee of Asan institute for Life Sciences (Seoul, Korea) and conformed to the US National Institutes of Health guidelines for humane handling of laboratory animals (IACUC-2020-12-189). The study was conducted in compliance with the ARRIVE guidelines. This study used 12 ETs in six pigs weighing 33.8–36.4 kg at 3 months. The six pigs were divided into two groups (i.e., the control and the SES groups) with three pigs in each group. The control group received an uncoated Co–Cr alloy stent, while the SES group received a sirolimus-eluting Co–Cr alloy stent. All pigs were supplied with water and food ad libitum and were maintained at a temperature of 24 °C ± 2 °C with a 12-h day–night cycle. Subsequently, all pigs were sacrificed 4 weeks after stent placement.

### Stent placement into the porcine ET and endoscopic examination

**Figure 3:**
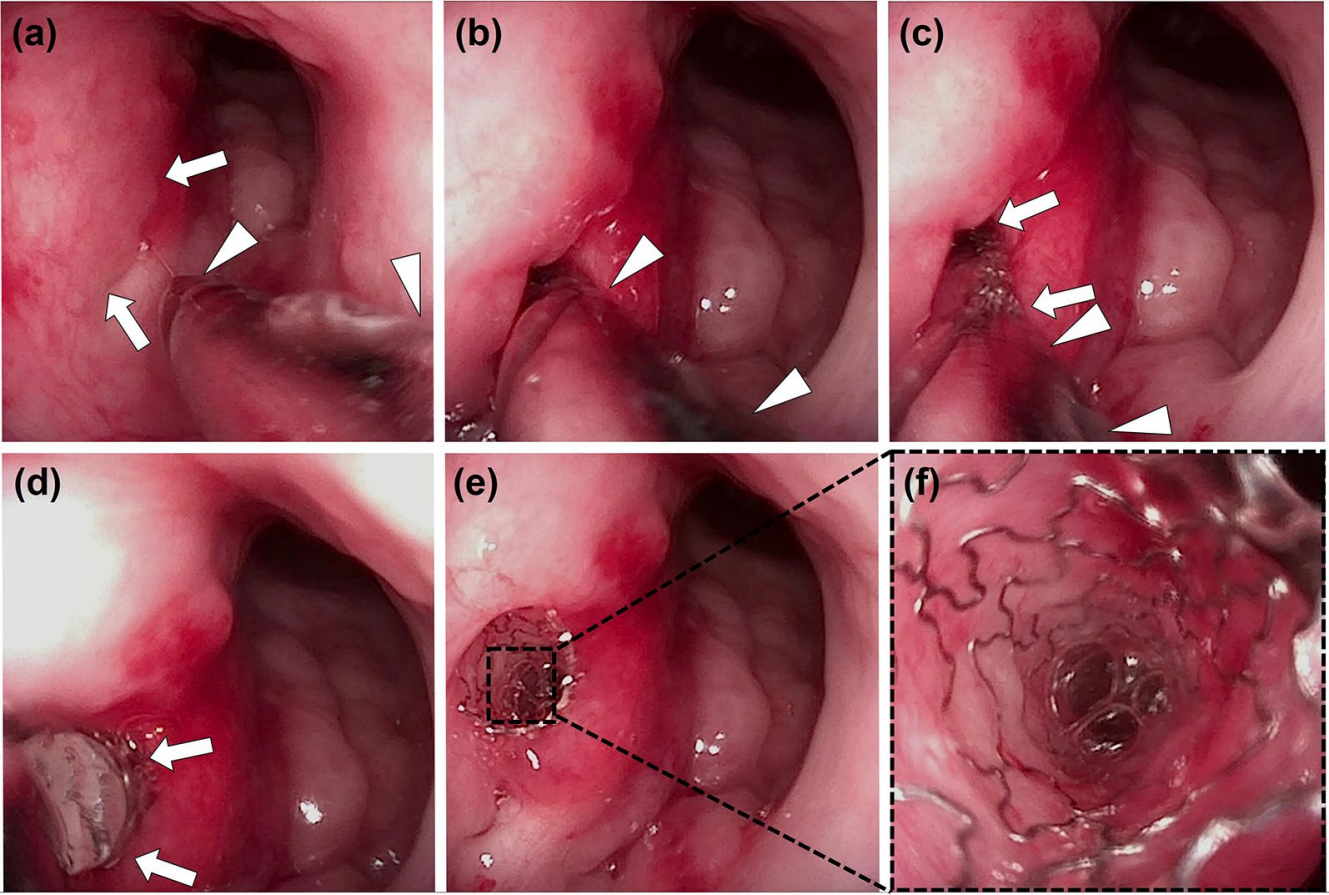
The technical steps of stent placement in a porcine Eustachian tube (ET) under endoscopic guidance. (**a**) Endoscopic image showing the nasopharyngeal orifice (*arrows*) and the inserted metallic guiding sheath (*arrowheads*). (**b**) The metallic guiding sheath (*arrowheads*) was inserted into the nasopharyngeal orifice. (**c**) The balloon catheter crimped with a stent (*arrows*) was inserted through the sheath (*arrowheads*) into the ET. (**d**) The balloon catheter (*arrows*) was fully inflated. (**e**) The proximal end of the stent protrudes from the nasopharyngeal ET orifice. (**f**) Endoscopic image showing luminal stent patency.

All pigs were anesthetized immediately before stent placement using a mixture of 50 mg/kg zolazepam, 50 mg/kg tiletamine (Zoletil 50; Virbac, Carros, France), and 10 mg/kg xylazine (Rompun; Bayer HealthCare, Leverkusen, Germany). An endotracheal tube was then placed, and anesthesia was administered by inhalation of 0.5–2% isoflurane (Ifran^®^; Hana Pharm. Co., Seoul, Korea) with 1:1 oxygen (510 mL/kg per min). The pig was in the prone position and baseline endoscopic examination (VISERA 4K UHD Rhinolaryngoscope; Olympus, Tokyo, Japan) was performed to check the nasopharyngeal orifice of the ET. The metallic guiding sheath was then advanced through the nostril to the nasopharyngeal orifice of the ET under endoscopic guidance (Fig. 3a,b). A balloon catheter, which was a crimped stent, was inserted through the sheath into the ET until its tip met resistance in the bony cartilaginous isthmus of the ET (Fig. 3c). The balloon catheter was fully inflated with saline to 9 atm as determined by a pressure gauge monitor (Fig. 3d). The balloon catheter was removed after stent placement (Fig. 3e), and the nasopharyngeal orifice was carefully evaluated for any procedure-related complications by endoscopy (Fig. 3f). All pigs underwent an endoscopic examination before and immediately after stent placement and 4 weeks after stent placement to evaluate the stent position with patency and the secretion presence around the stent.

### Histologic examination

All pigs were sacrificed with 75 mg/kg of potassium chloride via marginal ear vein injection. The head of the pigs was midsagittally sectioned using an electric saw, and the stented ET tissue samples were then carefully extracted for histologic examination (Supplementary Fig. 1a,b). The ET tissue samples were fixed in 10% neutral-buffered formalin for 24 h.

The ET tissue samples were sequentially dehydrated with alcohols of different concentrations. Samples were embedded in a resin block by infiltrations with glycol methacrylate (Technovit 7200^®^ VLC; Heraus Kulzer GMBH, Wertheim, Germany). Embedded ET tissue samples were axially sectioned at the proximal and distal portions (Supplementary Fig. 1c). The resin blocks were then mounted on acrylic slides. Using a griding system (Apparatebau GMBH, Hamburg, Germany), microgrinding and polishing of the resin block slides were performed with silicon carbide papers of different thicknesses until reaching 20 µm thick. All slides were stained with hematoxylin and eosin stains for histologic evaluation.

Histologic evaluations were performed to assess the tissue hyperplasia percentage, the submucosal fibrosis thickness, and the inflammatory cell infiltration degree. The percentage of tissue hyperplasia of ET cross-sectional area stenosis was calculated by solving the equation:

$$100 \times \left(1 - \frac{\text{Stenotic} \; \text{area} \; \text{of} \; \text{stent }({\text{mm}}^{2})}{\text{Original} \; \text{area} \; \text{of} \; \text{stent }({\text{mm}}^{2})}\right)$$

The thickness of submucosal fibrosis was vertically measured from the stent strut to the submucosal layer. The degree of inflammatory cell infiltration was subjectively determined according to the distribution and density of inflammatory cells, i.e., grade 1 (mild), visible occasional infiltration of single leukocytes; grade 2 (mild-to-moderate), patchy infiltration of leukocytes; grade 3 (moderate), coalescing leukocytes such that individual loci could not be distinguished; grade 4 (moderate-to-severe) diffuse infiltration of leukocytes throughout the submucosal layer; and grade 5 (severe), diffuse infiltration with multiple necrotic foci^28^. The thickness of submucosal fibrosis and degree of inflammatory cell infiltration were obtained by averaging eight points around the circumference. Histologic analysis of the ET was performed using a microscope (BX51; Olympus, Tokyo, Japan). Measurements were acquired using the CaseViewer software (CaseViewer; 3D HISTECH Ltd., Budapest, Hungary). The analysis of histological findings was based on the consensus of three observers blinded to the study.

### Statistical analysis

The Mann–Whitney *U* test was used to analyze the differences between the groups as appropriate. A *p* < 0.05 was considered statistically significant. A Bonferroni-corrected Mann–Whitney *U*-test was performed for *p* values < 0.05 to detect group differences (*p* < 0.008 as statistically significant). Statistical analyses were performed using SPSS software (version 27.0; SPSS, IBM, Chicago, IL, USA).

## Results

### Technical outcomes of stent placement into the porcine ET

Stent placement was technically successful in all pigs. Metallic guiding sheaths were successfully located in the nasopharyngeal orifice of the ET under endoscopic guidance although mucosal injury with touch bleeding was observed in four (33.3%) of the 12 specimens during the metallic sheath insertion. The touch bleeding was spontaneously resolved at 4 weeks of follow-up. All pigs survived until the end of the study without stent-related complications.

### Endoscopic findings

**Figure 4:**
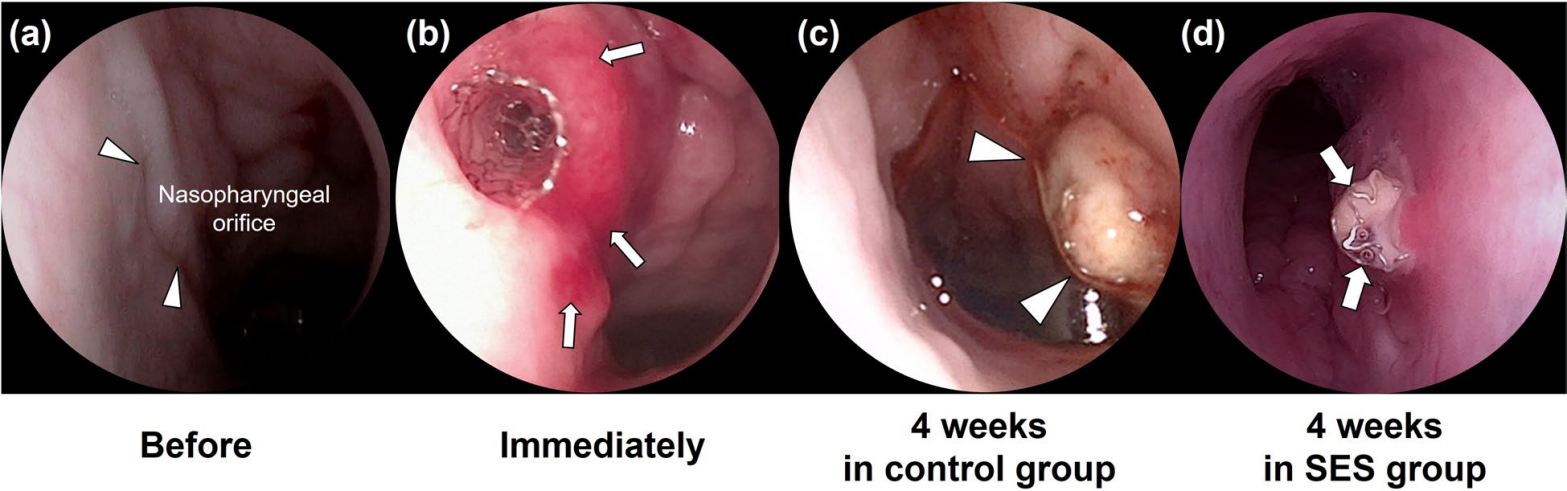
Endoscopic images of the porcine Eustachian tube (ET) in the control and sirolimus-eluting cobalt–chrome alloy stent (SES) groups. (**a**) Baseline endoscopic image was obtained before stent placement showing the nasopharyngeal orifice (*arrowheads*) of the ET. (**b**) Endoscopic image obtained immediately after stent placement showing the placed stent into the ET. Observed touch bleeding due to the metallic guiding sheath (*arrows*). (**c**) Endoscopic image obtained 4 weeks after stent placement showing mucus accumulation (*arrowhead*) around the stent. (**d**) Endoscopic image showing the stent that could not maintain the round shape (*arrows*).

The endoscopic findings are shown in Fig. 4. The stents were kept in place in all pigs at 4 weeks of follow-up study. Mucus accumulation was observed inside and around the stented ET in all (100%) ETs in the control group and three (50%) of six ETs in the SES groups, and the incidence rates did not differ between the two groups (*p* = 0.182). All placed stents were unable to keep the round shape.

### Histological findings

**Figure 5:**
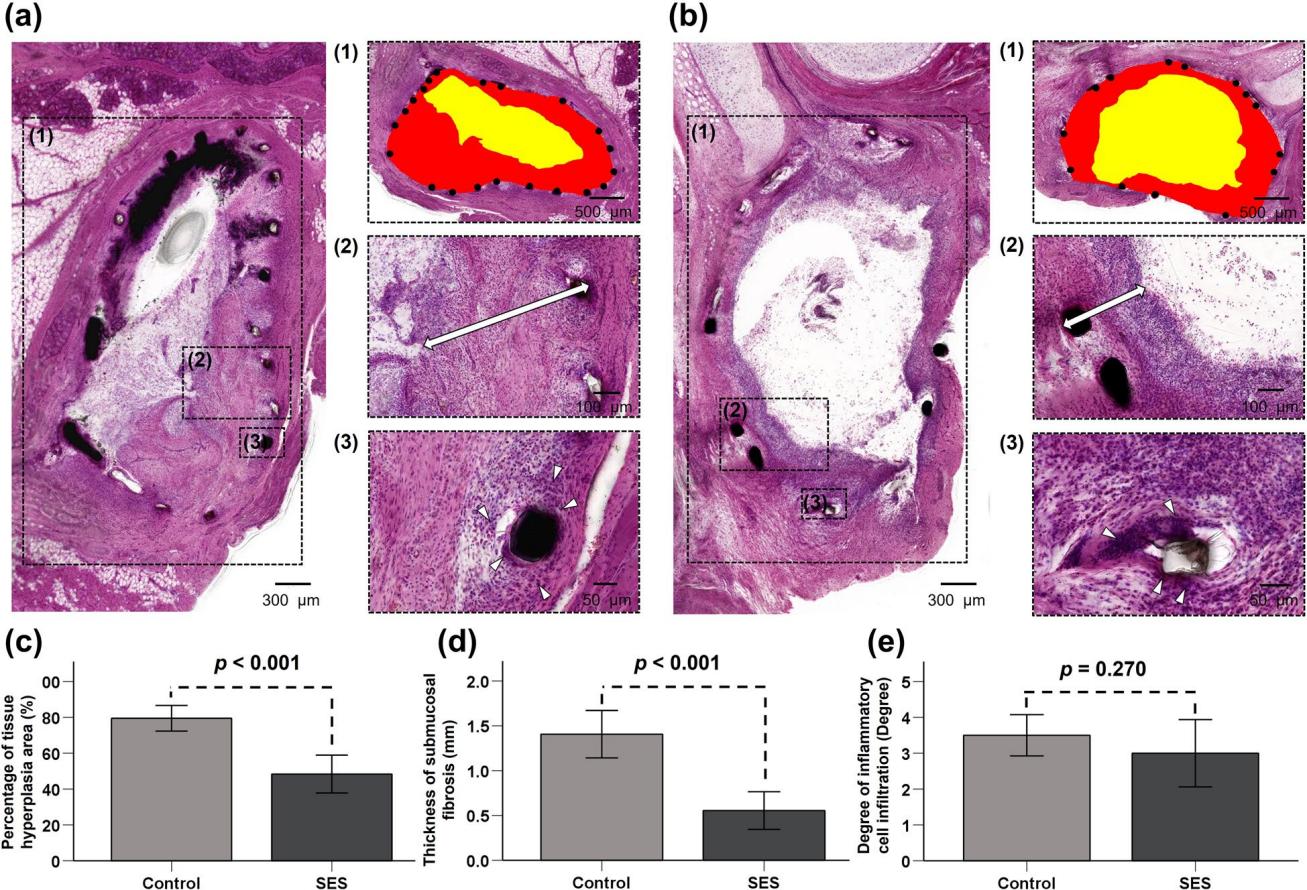
Analysis of histologic examinations for the stented Eustachian tube lumen in both groups. (**a**,**b**) The tissue hyperplasia area (*1* in **a** and **b**) and thickness of submucosal fibrosis (*2* in **a** and **b**; *double arrow*) were significantly larger in the control group than in the SES group, stent struts (*black dots*), stenotic lumen area (*yellow*), and original stented area (*red*). The degree of inflammatory cell infiltration (*3* in **a** and **b**; *arrowheads*) was not significantly different between the two groups. Histological results of (**c**) percentage of tissue hyperplasia area, (**d**) thickness of submucosal fibrosis, and (**e**) degree of inflammatory cell infiltration at 4 weeks after stent placement in both groups. SES, sirolimus eluting Co–Cr alloy stent.

The histological findings are shown in Fig. 5 and Supplementary Fig. 2. The tissue hyperplasia and the submucosal fibrosis proliferated between the stent strut in the ET lumen in both groups. The mean percentage of tissue hyperplasia area was significantly larger in the control group than in the SES group (79.48% ± 6.82% vs. 48.36% ± 10.06%, *p* < 0.001). Moreover, the mean thickness of submucosal fibrosis was also significantly higher in the control group than in the SES group (1.41 ± 0.25 vs. 0.56 ± 0.20 mm, *p* < 0.001). However, no significant difference in the degree of inflammatory cell infiltration was noted between the two groups (control group [3.50 ± 0.55] vs. SES group [3.00 ± 0.89], *p* = 0.270).

## Discussion

Drug-eluting stents have contributed to a high rate of stent patency, inhibiting stent-in-restenosis^20–24^. Stent-induced stenosis is caused by the formation of granulation tissue with fibrotic tissue changes in various nonvascular organs, including the esophagus, trachea, gastrodeodenum**,** and bile duct^29–33^. Pharmacologic agents such as dexamethasone, paclitaxel, gemcitabine, EW-7197, and sirolimus are coated onto the surface of stent wire meshes or covering membrane to prevent or treat tissue hyperplasia after stent placement^29,30,34–36^. More recent stent innovations via multi-functionalized stents using fusion technologies are actively investigated to treat nonvascular obstructive disorders^37–39^. In a previous study, stent-induced tissue hyperplasia was observed in the porcine ET model. Although stent development in the ET has not been sufficiently investigated, tissue responses after stent placement similar to those of other nonvascular luminal organs have been observed^19^. In the present study, SES was used to suppress stent-induced tissue hyperplasia in the porcine ET model. Sirolimus is toxic to islets and β-cell lines and can reduce cell viability and increase cell apoptosis^40,41^. Such effects may help suppress tissue hyperplasia formation by stimulating cell death. In our study, using the drug-eluting stent for the first time in the ET demonstrated to be effective in inhibiting the stent-induced tissue hyperplasia of the ET.

Balloon-expandable Co–Cr alloy stents used in the present study were readily available because it was commonly used in coronary arterial diseases^42^. Moreover, Co–Cr alloy has mechanical properties (e.g., high radial strength and nonelastic force)^43^. According to the endoscopic examination of the current study, Co–Cr alloy stents used for the porcine ET were unable to keep the round shape in all pigs due to insufficient elasticity without the ability to self-expand. The shape of the inserted stents may also conceivably be altered by movements surrounding the ET in a living animal (e.g., chewing and swallowing). The mechanical properties of Co–Cr alloy stents have become a disadvantage during stent placement in porcine ET. In addition, stent placement to the isthmus portion may lead to a permanently open ET. A permanently open ET, or a patulous ET, allows speech and nasopharyngeal sounds, a reflux from the gastrointestinal tract^44^, and pathogenic microorganisms^1^ to ascend into the middle ear, causing mucosal irritation and infections. Thus, the permanent nasopharyngeal opening should be prevented. Therefore, given the structure of the cartilaginous ET, it would be better if the stent is made of shape-memory alloy with superelastic properties (e.g., nitinol-based alloy). In general, severe secretion was detected inside and around the stent in the nasopharyngeal orifice. Accumulation of secretions in the stent protruding from the nasopharyngeal orifice is expected due to the blockage of the normal mucociliary movement of the mucus. Prevention of ascending infections to the middle ear is one of the main ET tasks^45^, and the placement method in which the stent protrudes out of the ET should be avoided because the direct stent contact with the bacterial flora in the nasopharynx may lead to increased ascending infection.

Balloon Eustachian tuboplasty via the nasopharyngeal orifice represents a novel, minimally invasive, therapeutic option for ET dysfunction that aims to open and expand the cartilaginous portion of the ET^8–10,46^. However, the underlying treatment mechanism has not yet been revealed^47^, and its long-term outcomes can be suboptimal^8,9,11,46^. In this case, temporary metallic stent placement can serve as an effective therapeutic option for patients who failed to respond to balloon Eustachian tuboplasty, and the feasibility of stenting in the ET has been verified by many preclinical studies. To assess in vivo tolerability and degradation profile, poly-l-lactide stents were implanted in chinchillas and rabbits through the tympanic membrane^17,18^. Moreover, a sheep model was established to evaluate the in vivo profile of a balloon-expandable metallic stent^15^. Our previous study developed a porcine ET model to investigate technical feasibility and assess stent-induced complications^19^, which laid a solid foundation for this study to examine the efficacy of SES using the previously established technique. In the present study, SES was successfully located in the cartilaginous portion to effectively suppress tissue hyperplasia. No stent-related complications occurred; however, mucosal injury with touch bleeding caused by the metallic guiding sheath was observed and resolved spontaneously within 4 weeks. Considering the possible complications from the metallic guiding sheath, improvement in the SES delivery system is urgent and crucial.

This study has some limitations. Although the histological findings reached significant differences between the groups, the number of animals in this study was too small to perform a robust statistical analysis. Although three observers conducted blinded analyses to evaluate interobserver variability, the degree of submucosal inflammatory cell infiltration was subjectively determined according to the distribution and density of inflammatory cells, as counting the inflammatory cells was difficult. Because our study was conducted using a limited number of large animals, a single dose of the drug was used, and in vivo pharmacokinetic study was not performed. Further studies should be performed to verify the optimal drug dosage and safety of sirolimus for the ET. Finally, the 4-week follow-up period was also a study limitation; thus, a study on the long-term efficacy of SES is needed.

The results of the present study demonstrated that SESs effectively suppressed tissue hyperplasia formation caused by mechanical injury after placing a balloon-expandable Co–Cr alloy stent in a porcine ET model. Stent-induced tissue hyperplasia-relative variables, including the tissue hyperplasia area and thickness of submucosal fibrosis, were significantly lower at 4 weeks after stent placement in the SES group than in the control group. SES appears to be effective in suppressing stent-induced tissue hyperplasia in the porcine ET. Although further investigation was required to verify the optimal stent materials and drug candidate with dosage, SES has localized therapeutic potential in preventing tissue hyperplasia in the ET after stent placement.

## Supplementary Information

Supplementary Figures.
