# Supplementary material for: Sirolimus-eluting cobalt–chrome alloy stent suppresses stent-induced tissue hyperplasia in a porcine Eustachian tube model
Source: Sci Rep. 2022 Mar 2;12:3436. doi: 10.1038/s41598-022-07471-2 (PMC8891339; doi:10.1038/s41598-022-07471-2)
Supplement: Supplementary file 1 — Supplementary Figures. [file 41598_2022_7471_MOESM1_ESM.docx]

SUPPLEMENTARY MATERIALS

**Sirolimus-eluting Cobalt-Chrome Alloy Stents Suppresses Stent-induced Tissue Hyperplasia in a Porcine Eustachian tube Model**

Jeon Min Kang, B.S.^1†^, Song Hee Kim, B.S.^1†^, Yeon Joo Choi, M.D.^2^, Yubeen Park, B.S.^1^, Dae Sung Ryu, B.S.^1^, Woo Seok Kang, M.D.^2^, Jung-Hoon Park, Ph.D.^1*^, Hong Ju Park, M.D.^2*^

*^1^Biomedical Engineering Research Center, Asan Institute for Life Sciences, Asan Medical Center, 88 Olympic-ro 43-gil, Songpa-gu, Seoul, 05505, Republic of Korea*

*^2^Department of Otorhinolaryngology-Head and Neck Surgery, Asan Medical Center, University of Ulsan College of Medicine, 88 Olympic-ro 43-gil, Songpa-gu, Seoul, 05505, Republic of Korea*

^†^J.M.K. and S.H.K. contributed equally to this work and are co-first authors.

^*^J.-H.P. and H.J.P. contributed equally to this work and are the co-corresponding authors.

**Correspondence:**

Jung-Hoon Park, Ph.D.^1^ and Hong Ju Park, M.D. Ph.D.^2^

^1^Biomedical Engineering Research Center, Asan Institute for Life Sciences, Asan Medical Center, 88 Olympic-ro 43-gil, Songpa-gu, Seoul, 05505, Republic of Korea

Tel: 82-2-3010-4123 Fax: 82-2-476-0090

E-mail: jhparkz[@amc.seoul.kr](mailto:hyjung@amc.seoul.kr)

^2^Department of Otorhinolaryngology-Head and Neck Surgery, Asan Medical Center, University of Ulsan College of Medicine, 88 Olympic-ro 43-gil, Songpa-gu, Seoul 05505, Republic of Korea

Tel: 82-2-3010-3700 Fax: 82-2-489-2773

E-mail: [dzness@hotmail.com](mailto:dzness@hotmail.com)


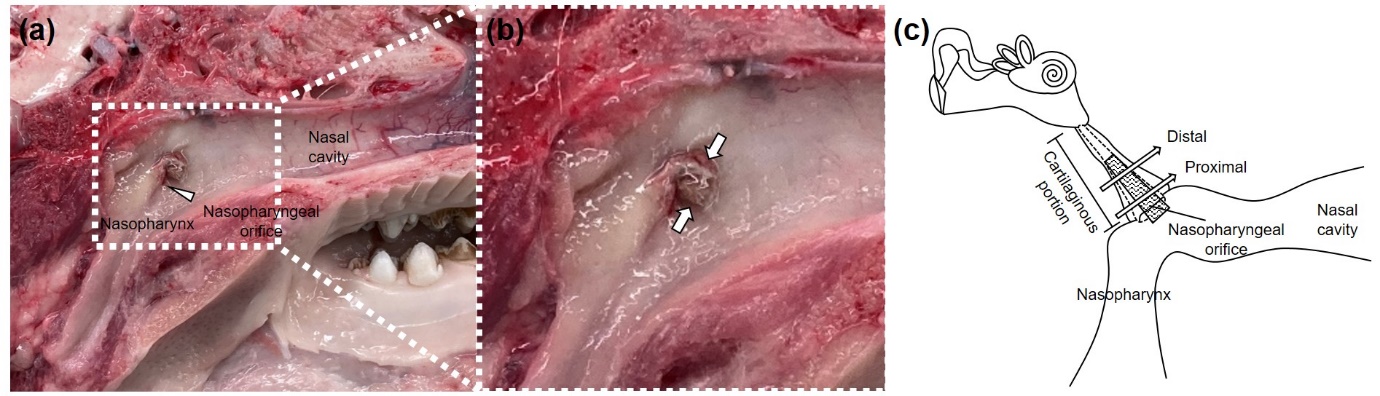


**Supplementary Figure 1.** Photographs of gross midsagittal sectioned porcine head and schematic illustration of the location of the stent into the Eustachian tube (ET) for histological examination. (**a**) The proximal end of the stent protruded from the nasopharyngeal orifice (*arrowhead*). (**b**) ET tissue sampling was sectioned together with a stent (*arrows*) inserted into the ET. (**c**) Stented ET samples were sectioned into two segments (*arrows*) at 5-mm intervals.


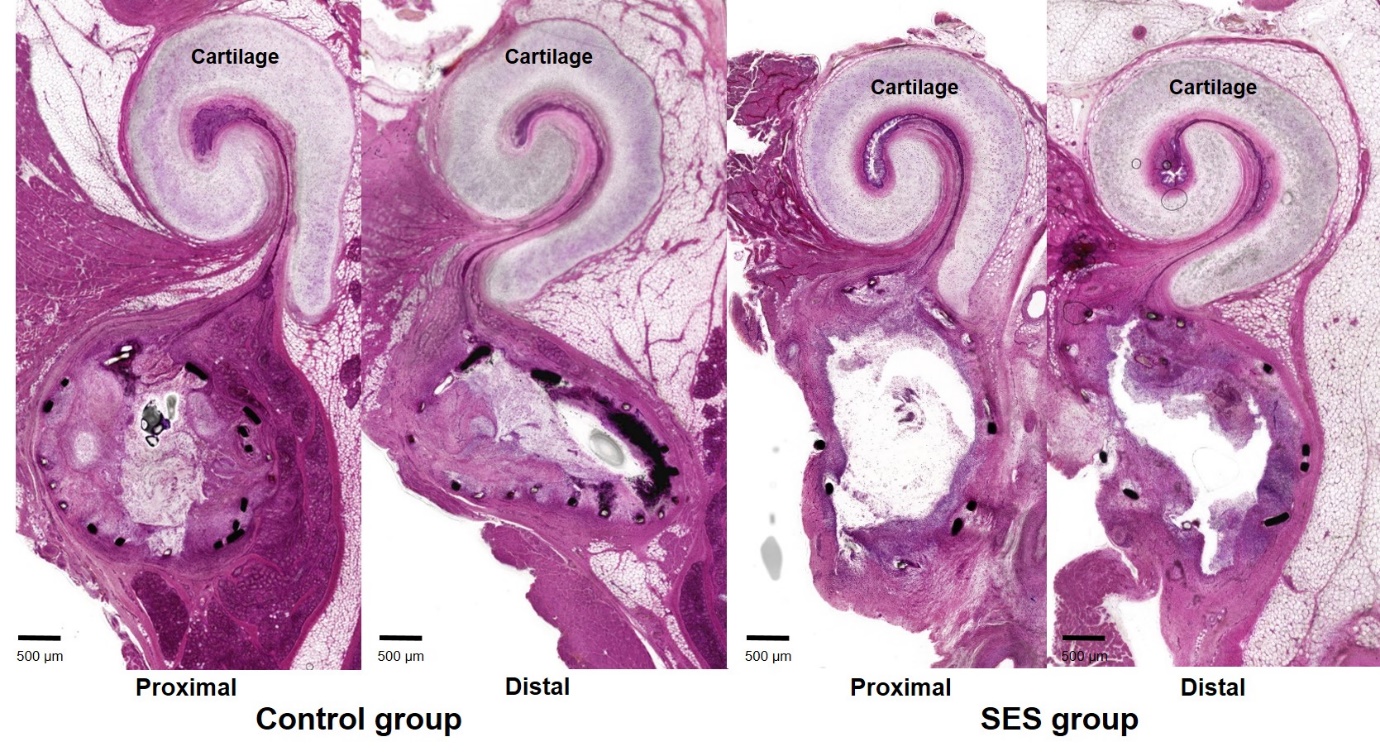


**Supplementary Figure 2** Representative microscopic images of histological sections obtained hematoxylin and eosin-stained sections at 4 weeks after stent placement.
